# “Just the Best Ones” combination: a new strategy for multi-epitope vaccine candidate based on immunoinformatics analysis to induce protective immunity against MRSA infection

**DOI:** 10.1128/spectrum.03085-25

**Published:** 2026-07-13

**Authors:** Qingru Chang, Lingdi Niu, Chuankun Zhang, Kun Xue, Xinran Yao, Hai Li, Zheng Jia, Fang Wang, Junwei Ge

**Affiliations:** 1 State Key Laboratory of Animal Disease Control and Prevention, Harbin Veterinary Research Institute, Chinese Academy of Agricultural Sciences 111613 , Harbin, China; 2 Heilongjiang Provincial Key Laboratory of Zoonosis, College of Veterinary Medicine, Northeast Agricultural University 12430, Harbin, China; University of Georgia College of Veterinary Medicine, Athens, Georgia, USA

**Keywords:** multi-epitope vaccine, MRSA, *Staphylococcus aureus*, immunoinformatics

## Abstract

*Staphylococcus aureus* is a major global health threat due to its increasing antibiotic resistance and high virulence, presenting serious challenges in healthcare. Currently, no effective vaccine exists to prevent its infections. In our study, we introduced a novel multi-epitope vaccine design method using immunoinformatics, named the “Just the Best Ones” strategy. This approach involved selecting the most promising T-cell and B-cell epitopes from 10 key proteins of *S. aureus* based on the pathogenic mechanism of the bacterium in the host, to create the *Staphylococcus aureus* Multi-Epitope Antigen (SAMEA) with optimal immunogenicity. Physicochemical and structural analyses confirmed SAMEA’s stability and suitable profiles for antigenicity, toxicity, and allergenicity. Molecular docking showed strong interactions between SAMEA and human TLR2, crucial for eliciting effective immune responses. Additionally, SAMEA was highly immunogenic and safe, generating a strong antibody response and excellent protection in mice. These results suggest that SAMEA is a promising multi-epitope vaccine candidate, highlighting the “Just the Best Ones” strategy’s effectiveness, and introducing a new direction in vaccine development against *S. aureus* infections.

**IMPORTANCE:**

Methicillin-resistant *Staphylococcus aureus* (MRSA) is a major health threat due to its antibiotic resistance and ability to cause severe infections in humans and animals. Yet, no licensed vaccine is available. Here, we present a “Just the Best Ones” strategy that applies immunoinformatics to select the most protective epitopes from key *S. aureus* antigens. Using this approach, we developed a multi-epitope antigen (SAMEA) that proved safe, highly immunogenic, and protective in mice, particularly when combined with a bacterium-like particle adjuvant. These results highlight SAMEA as a promising MRSA vaccine candidate and indicate that this strategy may help guide vaccine development for other difficult pathogens. As it continues to be refined, this approach could facilitate more effective vaccines and improve the prevention of infectious diseases.

## INTRODUCTION

*Staphylococcus aureus* is a major gram-positive pathogen that causes a broad spectrum of disease in both community and healthcare settings (1, 2). While typically a commensal organism, *S. aureus* can breach host barriers to cause diverse pathologies ranging from skin and soft tissue infections (SSTIs) (3) to life-threatening systemic conditions, including necrotizing pneumonia (4), endocarditis (5), meningitis (6), and sepsis (7). The mortality rate remains alarmingly high, reaching up to 50% in vulnerable populations such as infants in low-income regions (8). Beyond human morbidity, the global burden is further amplified by increasing reports of community-associated infections and the substantial costs associated with hospitalization and prolonged care (9, 10). Together, these features underscore the One Health relevance of *S. aureus* control. Beyond human health, *S. aureus* also represents an important threat to food safety and animal production (11). It is a leading cause of meat-borne food poisoning due to its production of staphylococcal enterotoxins (12), and it contributes to severe animal diseases across hosts, including poultry infections (13) and bovine mastitis (14), with associated productivity losses (15, 16) and potential zoonotic transmission (17).

Efforts to manage and prevent *S. aureus* infections encounter several obstacles. Current management relies heavily on antibiotics; however, the rapid emergence and spread of methicillin-resistant *S. aureus* (MRSA) and multidrug resistance across multiple antimicrobial classes increasingly limit therapeutic options (18–21). Compounding this issue is the fact that, unlike many bacterial infections, *S. aureus* typically fails to elicit a significant protective immune response. This means that individuals are often vulnerable to repeated *S. aureus* infections (22). Moreover, *S. aureus* possesses numerous virulence factors. Given the complex pathogenic nature of *S. aureus* (23, 24), which boasts an array of virulence factors, traditional vaccine strategies, which primarily induce humoral immune responses, have faced limitations in providing comprehensive immunoprotection (25). This is partly due to the bacterium’s ability to effectively evade host immune defenses (26). Effective treatment options for highly resistant *S. aureus* strains are extremely limited, thereby increasing the risk of treatment failure, particularly in severe infections and life-threatening situations. Collectively, these limitations make the exploration of alternatives to antibiotics an urgent public health need.

Over the past decades, multiple vaccine platforms have been explored, including inactivated, capsular polysaccharide (27), protein subunit (28), toxoid (29), and multivalent formulations (30). High-profile candidates targeting single or limited surface determinants—such as Merck’s V710 (targeting IsdB) (31) and Pfizer’s SA4Ag (targeting ClfA and MntC) (32)—faced obstacles in clinical trials due to concerns over efficacy and safety. Further, Pfizer’s tetravalent SA4Ag vaccine (33) and NovaDigm Therapeutics' NDV-3A vaccine (34), which targets podoplanar polysaccharide types 5 and 8, as well as the ClfA and MntC proteins, failed in preclinical trials. In addition, the five-antigen recombinant vaccine rFSAV demonstrated efficacy in phase 1a/1b clinical trials (35, 36). However, to date, no effective *S. aureus* vaccine has been commercialized (37).

Immunoinformatics-guided multi-epitope vaccines offer a promising alternative by enabling rational selection of epitopes to broaden population coverage and target multiple immune pathways. Such designs can, in principle, enhance the vaccine’s adaptability to *S. aureus* mutations and resistance, promising a stronger and more versatile immune response against the bacterium’s complex virulence factors. This paradigm has generated candidates for a range of pathogens, *Leishmania infantum* (38), *Mycoplasma synoviae* (39), *Mycobacterium tuberculosis* (40), *Coxiella burnetii* (41), and *Streptococcus* spp. (42). Nevertheless, for *S. aureus*, many multi-epitope studies remain largely computational, with limited *in vivo* validation (43–46). Moreover, there are notable limitations: many studies have focused on single antigens or a few antigens, such as staphylococcal protein A (SpA) (47, 48), Clumping factor surface proteins (ClfA and ClfB) (45, 49, 50), and Fn-binding proteins (FnbpA and FnbpB) (46). This approach does not cover the different stages of pathogen invasion, limiting the vaccines to elicit protective immune responses against all stages of infection (51). Conversely, expanding antigen selection can inflate epitope number and increase immunological “burden,” raising practical challenges in construct design and immune focusing.

**Fig 1:**
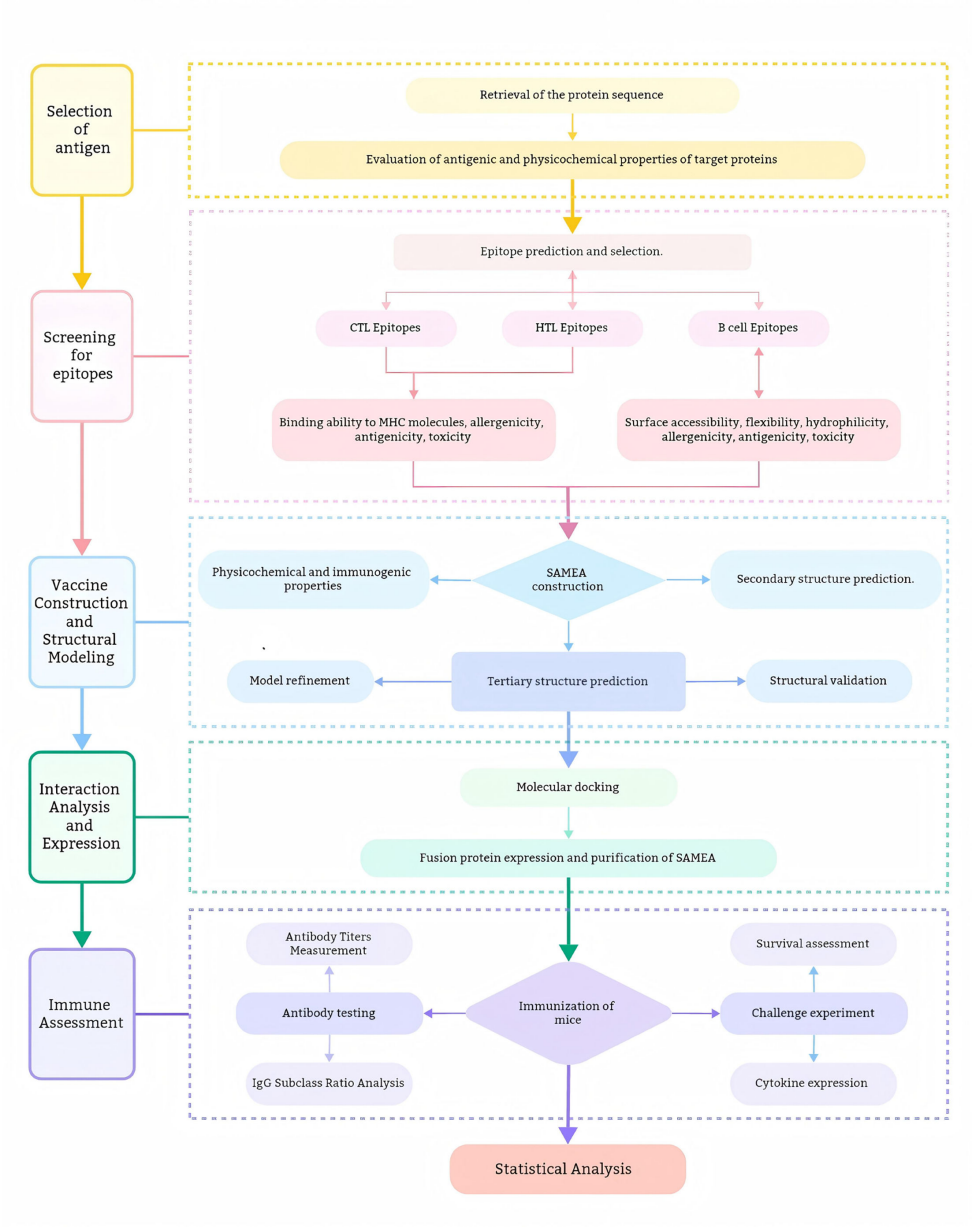
Schematic representation of the workflow for the development of SAMEA against MRSA infections.

In response to these challenges, our study introduces a novel vaccine construction strategy, “Just the Best Ones,” which utilizes immunoinformatics to meticulously select and combine the optimal immunogenic epitopes. Through this strategy, we developed a *S. aureus* Multi-Epitope Antigen (SAMEA) to protect against *S. aureus*. To ensure the efficacy and stability of the vaccine, SAMEA was evaluated for its physicochemical properties, antigenicity, and structure, as well as its interaction with TLR receptors. After evaluating the indicators, we conducted animal experiments using various immunological assays. These included measurements of total IgG, IgG1, IgG2a, different Th1 and Th2 cytokines, as well as protection assays against MRSA challenges and assessments of bacterial burden to evaluate the protective effects on mice. Figure 1 shows the overall workflow used in this study. The *in vivo* experiments highlight the vaccine’s safety and immunogenicity in combating *S. aureus*. Overall, we propose a strategy to design multi-epitope vaccines not only for *S. aureus* but also for other pathogens with complex immune evasion mechanisms.

## MATERIALS AND METHODS

### Bacterial strains and animals

The MRSA strain*,* USA-300 (ATCC BAA-1717), was spread on TSB agar (AOBOX, Beijing, China), and incubated overnight at 37°C for 18 h. Single colonies were picked and inoculated into TSB liquid medium (AOBOX). After 12 h of incubation at 37°C on a shaker at 220 rpm, the bacteria were washed and resuspended with PBS, and the concentration of the bacterial suspension was adjusted to 10^9^ CFU/200 μL.

Female Kunming mice (SPF class) aged 4–6 weeks and weighing 18–22 g were purchased from the Liaoning Changsheng Biotechnology Co., Ltd. (Shenyang, China).

### Antigen selection and sequence retrieval

Previous studies have demonstrated that *S. aureus* pathogenesis involves multiple stages, including adhesion, invasion, immune evasion, and toxin-mediated damage (52), which together determine infection establishment and progression. Based on these pathogenic characteristics and previous vaccine design studies (25, 53, 54), candidate antigens were selected according to three key criteria: (i) key virulence factors involved in different infection stages (adhesion, invasion, and evasion); (ii) high surface accessibility, ensuring optimal exposure to the host immune system; and (iii) high degree of conservation across major MRSA clinical isolates. Accordingly, 10 representative proteins were selected as the template for SAMEA (Table 1).

**TABLE 1:** Selection and functional characterization of the 10 target antigens in SAMEA

Antigen	Functional category	Role in pathogenesis	References
ClfA	Adhesion, invasion, and biofilm formation	Binds fibrinogen to mediate attachment to host tissues and implanted devices. Immune evasion. Promotes clot-associated colonization, biofilm formation, and persistence in bloodstream infections.	(55–57)
FnbPA	Adhesion, invasion, and biofilm formation	Mediates adhesion to ECM proteins, induces integrin-dependent invasion, promotes biofilm formation and platelet aggregation, contributes to endocarditis and dissemination.	(58, 59)
IsdB	Adhesion, nutrient acquisition, and invasion	Acts as a high-affinity surface receptor for hemoglobin to extract iron; promotes adhesion to host cells; triggers TLR4–MyD88–NF-κB signaling and NLRP3 inflammasome activation to induce pro-inflammatory cytokines.	(60–62)
PNSG	Biofilm formation	Major biofilm exopolysaccharide that promotes intercellular adhesion and protects bacteria from phagocytosis and complement-mediated killing, contributing to chronic and device-related infections.	(63–65)
SpA	Adhesion and immune evasion	Acts as a multifunctional surface virulence factor that interferes with host humoral immunity and promotes bacterial adhesion to host tissues, thereby facilitating immune evasion and the development of invasive infections.	(66, 67)
sasX	Adhesion, colonization, biofilm formation, and immune evasion	Promotes nasal colonization, bacterial aggregation and biofilm formation, enhances immune evasion, and significantly increases virulence in skin and lung infection models.	(68)
Hla	Invasion and toxin	Acts as a pore-forming cytotoxin that induces host cell lysis, disrupts epithelial and endothelial barriers, promotes inflammation, and significantly contributes to tissue damage and disease severity.	(69, 70)
MntC	Adaptation and nutrient acquisition	Acts as the manganese-binding component of the MntABC transporter to support oxidative stress resistance and is required for full virulence during infection.	(71, 72)
SEB	Toxin (superantigen)	Acts as a potent superantigen that crosslinks MHC class II molecules and T-cell receptors, leading to massive polyclonal T-cell activation, cytokine storm, acute gastrointestinal inflammation, toxic shock, and potential systemic organ injury.	(73–75)
Plc	Invasion and virulence	Enhances bacterial invasiveness and immune evasion by cleaving GPI-anchored proteins, generating diacylglycerol, disrupting host membranes, modulating immune signaling, and being consistently expressed in pathogenic *S. aureus* strains.	(76, 77)

Using *S. aureus* (Acc. NCTC 8325) as the reference strain, the amino acid sequences of these ten antigens were retrieved from the National Center for Biotechnology Information (NCBI) database (https://www.ncbi.nlm.nih.gov/) in FASTA format (78). The selected proteins include: clumping factor (ClfA), Fn-binding proteins A (FnBPA), alpha-hemolysin (Hla), iron-regulated surface determinant B (IsdB), manganese transporter protein C (MntC), phosphatidylinositol phosphodiesterase C (Plc), poly-N-succinyl beta-1-6 glucosamine (PNSG), staphylococcal surface protein (sasX), staphylococcal enterotoxin B (SEB), and staphylococcal protein A (SpA). These selected antigens collectively represent multiple functional categories involved in *S. aureus* pathogenesis, including adhesion (e.g., ClfA, FnBPA, and sasX), toxin-mediated damage (e.g., Hla and SEB), immune evasion (e.g., SpA), and nutrient acquisition (e.g., IsdB and MntC). This functional diversity ensures that the selected antigen set provides broad coverage of key pathogenic mechanisms across different stages of infection.

To ensure these candidates possessed suitable molecular stability for vaccine development, the ExPASy server’s ProtParam tool (https://web.expasy.org/protparam/) was used to characterize all of the proteins' functional physicochemical characteristics (79). Parameters such as molecular weight, theoretical pI, instability index, and grand average of hydropathicity (GRAVY) were analyzed to provide a baseline for the subsequent multi-epitope design.

Unlike conventional multi-epitope vaccine designs that either focus on a limited number of antigens or include multiple epitopes per antigen, our approach prioritizes broad antigen coverage while restricting epitope selection to only the highest-scoring candidate from each antigen. This strategy aims to balance comprehensive immune coverage with reduced epitope redundancy and immunological burden.

### Epitopes prediction

Several B-cell epitope prediction systems, including BepiPred-2.0 (80), Emini Surface Accessibility Prediction tool (81), Karplus and Schulz flexibility tool (82), and Parker Hydrophilicity Prediction method (83) of IEDB (http://tools.iedb.org/bcell), were utilized to predict B-cell epitopes for vaccine development. These B-cell epitopes were selected based on a combination of high binding affinity to MHC molecules, high antigenicity scores above a VaxiJen threshold of 0.4, absence of allergenicity according to AllerTOP, and non-toxicity as per ToxinPred evaluation, ensuring the chosen epitopes have the greatest potential for an effective immune response. CTL epitopes were identified by submitting the FASTA sequences of the ten target proteins to the NetCTL-1.2 server (https://services.healthtech.dtu.dk/services/NetCTL-1.2/). A threshold of 0.75 corresponds to sensitivity and specificity of 0.80 and 0.97, respectively (84). To predict epitopes capable of binding to the MHC II molecule, the NetMHCIIpan 4.0 server (https://services.healthtech.dtu.dk/service.php?NetMHCIIpan-4.0) was used (85). Then, all epitopes identified as MHC ligands were chosen for additional investigation. Protective antigens were predicted using the VaxiJen server (http://www.ddg-pharmfac.net/vaxijen/) (86). Epitopes that fell short of the 0.4 threshold level set by the VaxiJen server were eliminated. Additionally, the allergenicity of chosen epitopes was evaluated using AllerTOP (https://www.ddg-pharmfac.net/AllerTOP/) (87), and the toxicity was evaluated using the ToxinPred webserver (https://webs.iiitd.edu.in/raghava/toxinpred/) (88). Epitopes that are antigenic, non-toxic, and non-allergenic were chosen to create longer pieces by combining adjacent and overlapping epitopes (89).

### Vaccine design and physicochemical and immunogenic properties prediction

Highly scored peptides that can cover multiple MHC super-types are screened as “the best” epitopes and linked by appropriate fusion protein linkers to design the multi-epitope vaccine. Three linkers, KK, GGGS, and GPGPG, as described (90), were utilized to link B-cell epitopes, CTL epitopes, and HTL epitopes, respectively. The antigenicity and sensitization of the vaccine constructions were assessed using the VaxiJen 2.0 server and the AllerTOP server, and the physical and chemical properties of the vaccines were calculated using ProtParam of ExPASy. Finally, the vaccine constructions were uploaded to the SOLpro server (http://scratch.proteomics.ics.uci.edu/) to be tested for solubility (91).

### Structure prediction, refinement, validation, and molecular docking calculation

Secondary and tertiary structure predictions were performed by using the PSIPRED (92) (http://bioinf.cs.ucl.ac.uk/psipred/). Tertiary structure prediction was performed using the I-TASSER server (93) (https://zhanggroup.org/I-TASSER/), a well-established and widely validated platform that integrates threading, *ab initio* modeling, and iterative structural assembly. Given that SAMEA is an artificially constructed fusion protein composed of multiple epitopes connected by flexible linkers, I-TASSER provides a suitable framework for modeling such chimeric sequences, enabling accurate prediction of both structured epitope regions and unaligned linker regions. To improve the accuracy of the anticipated 3D modeled structure, the MoD Refiner server (https://zhanggroup.org//ModRefiner/) was utilized (94). PROCHECK was also used to examine the redesigned vaccine construct’s remaining geometry and forecast its improved stereochemical quality for validation (95).

The TLR2 complexes (PDB ID: 6NIG) were retrieved from the protein data bank (RCSB) (96) with a resolution of 2.35 Å. Prior to molecular docking, PyMOL was used to process TLR2 and the vaccine. To test the developed vaccine’s binding affinity to TLR molecules, it was docked at the designated receptors using the ClusPro 2.0 server (https://cluspro.org/) (97). Based on root-mean-square deviation (RMSD), the numerous lowest energy structures produced by ClusPro were clustered, and the biggest clusters—representing the most plausible complicated models—were discovered. Using PyMOL software, the docked structure and vaccine-receptor interaction were visualized.

Molecular dynamics simulations were conducted using Gromacs 2022.3 software. Molecules were preprocessed with AmberTools22 to incorporate the GAFF force field. Hydrogenation of molecules and RESP potential calculations were performed using Gaussian 16W. The simulations were maintained at a constant temperature of 300 K and atmospheric pressure (1 bar). The Amber99sb-ildn force field was employed, using Tip3p water model for the solvent. System charge neutrality was achieved by adding an appropriate number of Na^+^ ions. Energy minimization was performed using the steepest descent method, followed by equilibration in the isothermal-isochoric (NVT) and isothermal-isobaric (NPT) ensembles for 100,000 steps each, with a coupling constant of 0.1 ps and a duration of 100 ps. Subsequent free molecular dynamics simulations were executed over 5,000,000 steps with a timestep of 2 fs, totaling 100 ns. Post-simulation, trajectory analysis was conducted using the built-in tools of the software to calculate the root-mean-square deviation (RMSD), root-mean-square fluctuation (RMSF), and the radius of gyration for each amino acid in the protein.

### Expression and purification of the fusion protein

The SAMEA gene was successfully synthesized by Comate Bioscience (Changchun, Jilin, China) and was ligated into the pET24a vector using restriction sites *Bam*HI and *Nde*I. To express and purify the SAMEA protein, the pET24a-SAMEA plasmid was transformed into the *E. coli* BL21 (DE3) competent cells. The transformed cells were plated onto LB agar containing kanamycin and incubated at 37°C for 14–18 h. Positive single colonies were selected and inoculated into LB liquid medium supplemented with kanamycin, followed by incubation at 37°C with shaking at 180 rpm. When the bacterial culture reached an optical density (OD_600_) of 0.6–0.8, protein expression was induced by adding isopropyl-β-d-thiogalactopyranoside (IPTG) to a final concentration of 0.5 mM. The culture was then incubated at 25°C with shaking at 180 rpm for 24 h to promote recombinant protein expression. The protein expression was analyzed by SDS-polyacrylamide gel electrophoresis (SDS-PAGE) as previously described (98). The fusion protein was purified using the His-tag Purification Resin (Beyotime, Shanghai, China) following the manufacturer’s protocol. For Western blot analysis, proteins separated by SDS-PAGE were transferred to a PVDF membrane, blocked, and washed with PBST. The HRP-labeled mouse anti-His-tag monoclonal antibody was incubated and washed. Finally, the ECL chemiluminescent substrate kit, Liquid A, and Liquid B were mixed in equal volumes and added dropwise onto the PVDF membrane, ensuring that the entire PVDF membrane was infiltrated, and observed using a gel imaging analyzer.

### Immunization of mice

#### Single-dose immunization

Forty mice were randomly divided into four groups (*n* = 10 per group) after adapting to the environment for 7 days (41), with randomization performed using a computer-generated random sequence. On day 0, the control group received an intraperitoneal injection of 200 μL of PBS. Meanwhile, the three immunized groups were administered 200 μL of the SAMEA vaccine, SAMEA combined with alum, or SAMEA and the adjuvant pBLP, respectively. The pBLP, derived from *Listeria monocytogenes*, was prepared as in our previous report (99), with each mouse in the pBLP group receiving 200 μL 1U during immunization. The order of immunization, measurement, and analysis was consistent for all groups to minimize potential confounding factors.

#### Two-dose immunization

On day 0, the control group was injected with PBS, and each of the three immunized groups was vaccinated. On day 21, mice were immunized for a second time, consistent with day 0. The immunization procedure and dosage were the same as in the single immunization experiment.

Clinical responses were observed daily after the immunization. The mice were then challenged intraperitoneally with a lethal dose of MRSA USA300, 50 μL (10^9^ CFU/200 μL), 21 days after the final immunization to cause a systemic infection. Continuous monitoring was conducted to track the survival of the mice. Concurrently, their weights were recorded. Mice blood samples were collected on days 7, 14, and 21 after the initial immunization (and additionally on days 28, 35, and 42 for the two-dose immunization group) for the subsequent detection of the relevant indices. Mice were euthanized by cervical dislocation 21 days after infection, and kidneys were collected. Kidneys of mice were used for bacterial count analysis as described previously (100).

### Detection of antibodies

Consistent with previously used methods (101), the endpoint method was used in this experiment. ELISA plates were coated overnight at 4°C with SAMEA antigen at 3 μg/mL. After washing and blocking, serially diluted serum samples were added and incubated. HRP-conjugated secondary antibodies were then applied, followed by TMB substrate development. The reaction was stopped, and absorbance was measured at 450 nm. For antibody avidity analysis, 4 M urea was added after serum incubation to remove low-affinity antibodies, and the remaining steps were performed as described previously (102).

### Bacterial counts

Bacterial counts were performed on mouse kidney tissues in each group, and 10 times the volume of tissue was added to the homogenization tube with sterile PBS, and the tissues were added to the homogenization tube and ground completely. A 10-fold gradient was used to dilute 100 μL of tissue homogenate until it reached a dilution of 10^−8^ times the original concentration. Then, 50 μL of each dilution was coated on a TSA solid plate and incubated overnight at 37°C. Colonies on the plate were counted 18 h later.

### Real-time PCR for cytokines in the mouse kidney

The expressions of cytokines were quantified by real-time PCR (qRT-PCR). Briefly, total RNAs were extracted from kidneys of all mice in each group using TRIzol (Invitrogen, Sigma-Aldrich, St. Louis, MO, USA). cDNA was generated with random primers. Applied Biosystems 7500 Real-Time PCR Systems were used for the qPCR experiments. The tests were carried out to detect the expression of IL-1β, IL-6, IFN-β, TNF-α, TLR2, and TLR4, following the manufacturer’s instructions. The relative expression levels were calculated using the 2^−∆∆CT^ method.

### Statistical analysis

Data are presented as mean ± SD. Statistical analyses were performed in SPSS 19.0. For comparisons among multiple groups, one-way ANOVA was used, followed by Tukey’s post hoc test. Survival curves were compared using the log-rank (Mantel–Cox) test. Where indicated, significance is shown as *P < 0.05, **P < 0.01, and ***P < 0.001. Graphs were generated in GraphPad Prism 9 (GraphPad Software, La Jolla, CA, USA).

## RESULTS

### Successful prediction of epitopes and SAMEA construction

The amino acid sequences of key *S. aureus* proteins were retrieved from the NCBI database. As outlined in Table 2, each protein’s critical physicochemical properties, including molecular weight, aliphatic index, theoretical pI, grand average of hydropathicity (GRAVY), and instability index, were analyzed. These physicochemical parameters were further considered during epitope screening and served as a baseline for evaluating the final multi-epitope construct.

**TABLE 2:** Physiochemical parameters of a target protein of *S. aureus*

Protein name	Number of amino acids	Molecular weight (Da)	Theoretical pI	Instability index	Aliphatic index	Grand average of hydropathicity
ClfA	1,093	113,034.56	3.43	54.67	39.97	−1.206
FnbPA	572	62,855.36	4.73	29.01	66.89	−0.803
IsdB	107	12,546.29	6.29	49.36	71.96	−0.566
PNSG	350	41,600.52	8.20	26.03	132.26	0.807
SpA	524	57,320.13	5.54	50.35	61.95	−1.191
sasX	204	21,122.85	5.88	18.46	66.08	−0.598
Hla	319	35,961.33	8.69	21.72	69.03	−0.662
MntC	312	35,071.05	8.67	25.67	78.72	−0.723
SEB	266	31,435.87	8.65	34.00	74.66	−0.664
Plc	193	21,787.17	7.70	23.08	64.72	−0.708

**Fig 2:**
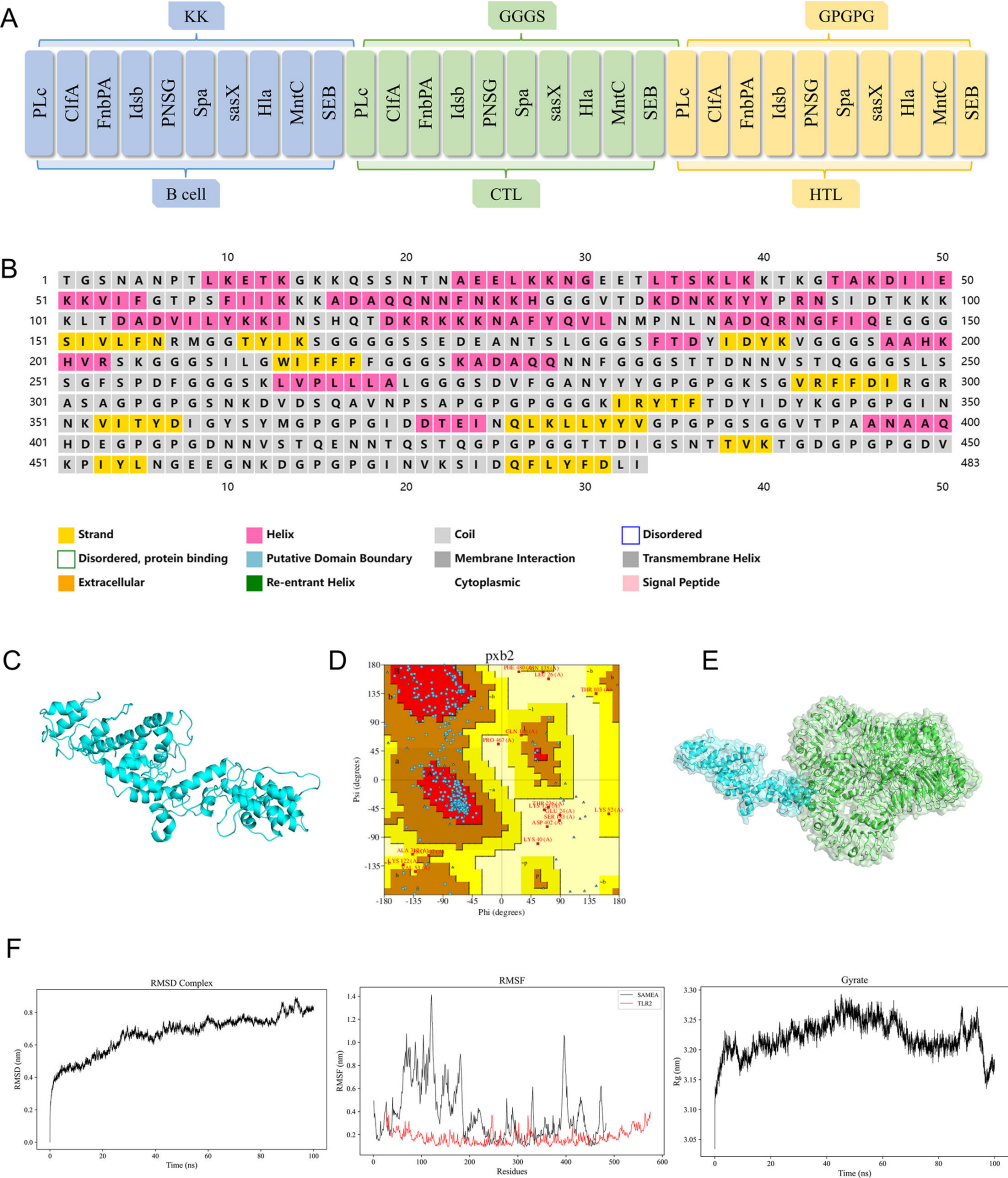
Construction and evaluation of SAMEA. (**A**) Schematic architecture of the final multi-epitope construct, showing the arrangement of B-cell, CTL, and HTL epitopes connected by linkers (KK, GGGS, and GPGPG). (**B**) Predicted secondary-structure content of SAMEA. (**C**) Predicted tertiary structure of SAMEA. (**D**) Ramachandran plot of the refined model. (**E**) Docked complex of SAMEA (cyan) with TLR2 (green). (**F**) Molecular dynamics simulation of the SAMEA–TLR2 complex showing root-mean-square deviation (RMSD), root-mean-square fluctuation (RMSF), and radius of gyration (Rg).

Building on this foundational data, we submitted the sequences of these ten proteins to Bepipred for antigenicity analysis. The resulting data, in conjunction with assessments of the predicted epitopes' allergenicity and toxicity, refined our selection process. After adjusting for epitope overlaps, we identified 10 B-cell epitopes that met our rigorous criteria, as detailed in Table 3. We then employed NetCTL 1.2 to calculate the composite scores of the selected epitopes, and after appropriate screening, we chose 10 potential CTL epitopes (Table 4). Additionally, 10 HTL epitopes with low percentile ranks were chosen, as reflected in Table 5. In the SAMEA construction, we strategically selected one optimal B-cell, CTL, and HTL epitope from each antigen (Fig. 2A). This approach resulted in a total of 30 epitopes. The SAMEA formulation phase involved strategically assembling the vaccine by integrating the most promising B-cell, CTL, and HTL epitopes, linked via KK, GGGS, and GPGPG. The final SAMEA design has 472 amino acid residues (Supplemental material).

**TABLE 3:** Potential final BCL epitopes from target proteins of *Staphylococcus aureus*

Epitopes	Protein	Start position	End position	Karplus and Schulz flexibility prediction results	Parker hydrophilicity prediction results
TGSNANPTLKETKG	Plc	172	185	1.046	3.659
QSSNTNAEEL	ClfA	145	154	1.081	5.482
NGEETLTSK	FnbPA	130	136	1.069	4.167
TKGTAKDIIE	IsdB	12	20	1.071	3.748
VIFGTPSFII	PNSG	382	388	1.012	3.746
KKNAFYQVLNMPNLNADQRNGFIQE	SpA	61	85	1.057	3.9
HGGGVTDKDN	sasX	94	99	1.044	5.125
YYPRNSIDTK	Hla	128	137	1.053	3.054
KLTDADVILY	MntC	79	88	1.000	1.115
INSHQTDKRK	SEB	130	139	1.033	5.354

**TABLE 4:** Potential final CTL epitopes from target proteins of *Staphylococcus aureus*

Epitope	Protein	Super type	Antigenic score	Allergenicity	Toxicity
IVLFNRMGGTYIKSG	Plc	A1, A26, and B62	1.4767	Probable non-allergen	Non-toxin
SEDEANTSL	ClfA	B39 and B44	1.1827	Probable non-allergen	Non-toxin
FTDYIDYKV	FnbPA	A1 and A2	0.6058	Probable non-allergen	Non-toxin
AAHKHVRSK	IsdB	A3 and A26	1.7868	Probable non-allergen	Non-toxin
ILGWIFFFF	PNSG	A3, A26, B58, and B62	3.9617	Probable non-allergen	Non-toxin
KADAQQNNF	SpA	A1 and A58	1.2114	Probable non-allergen	Non-toxin
TTDNNVSTQ	sasX	A1	2.2329	Probable non-allergen	Non-toxin
LSSGFSPDF	Hla	A1, A58, and B62	1.1149	Probable non-allergen	Non-toxin
KLVPLLLAL	MntC	B8 and B62	1.2616	Probable non-allergen	Non-toxin
DVFGANYYY	SEB	A1, A26, and B62	1.1312	Probable non-allergen	Non-toxin

**TABLE 5:** Potential final HTL epitopes from target proteins of *Staphylococcus aureus*

Epitope	Protein	Allele	Antigenic score	Allergenicity	Toxicity
KSGVRFFDIRGRASA	Plc	DRB5_0202	0.9818	Probable non-allergen	Non-toxin
SNKDVDSQAVNPSAP	ClfA	DRB1_0403	1.2432	Probable non-allergen	Non-toxin
GGKIRYTFTDYIDYK	FnbPA	DRB1_0701 and DRB4_0101	1.2157	Probable non-allergen	Non-toxin
INNKVITYDIGYSYM	IsdB	DRB3_0101	0.5662	Probable non-allergen	Non-toxin
IDDTEINQLKLLYYV	PNSG	DRB1_1201	0.9512	Probable non-allergen	Non-toxin
SGGVTPAANAAQHDE	SpA	DRB1_0401, DRB1_0402, DRB1_0403, DRB1_0404, and DRB1_0408	0.8381	Probable non-allergen	Non-toxin
DNNVSTQENNTQSTQ	sasX	DRB1_0404, DRB4_0101, and DRB4_0103	1.1959	Probable non-allergen	Non-toxin
GTTDIGSNTTVKTGD	Hla	DRB3_0202	1.1438	Probable non-allergen	Non-toxin
DVKPIYLNGEEGNKD	MntC	DRB3_0202	1.7319	Probable non-allergen	Non-toxin
INVKSIDQFLYFDLI	SEB	DRB1_1201	1.1741	Probable non-allergen	Non-toxin

After that, the developed SAMEA was subjected to additional physicochemical and immunogenic assessments (Table 6). To verify compatibility, SAMEA was analyzed against the human proteome, which confirmed the absence of significant similarities. Evaluations of allergenicity and antigenicity suggested a likely non-allergenic nature and revealed a high level of antigenicity with a score of 1.3413. SAMEA possesses a theoretical isoelectric point (pI) of 7.84, implying that it will exhibit a net zero charge at a near-neutral pH. This characteristic is crucial as it may influence its solubility and the efficacy of its interaction with immune cells. With a GRAVY value of −0.720, indicating SAMEA’s hydrophilic nature, this property significantly enhances its solubility in bodily fluids and promotes more effective distribution and interaction with the immune system. Additionally, its thermal stability and a molecular weight of 49.089 kDa place it in a range favorable for efficient processing and presentation by the immune system. These properties demonstrate the high immunogenicity potential of SAMEA, proving its suitability for further experimental studies.

**TABLE 6:** Physicochemical properties, antigenic, immunogenic, allergenic, and solubility of the SAMEA^*a*^

Parameter	Value	Remark
Number of amino acids	472	–
Molecular weight	49,089.25	Moderate
Theoretical pI	7.84	Basic
Total number of atoms	6,828	–
Estimated half-life	7.2 h >20 h >10 h	Mammalian reticulocytes, *in vitro* Yeast, *in vitro* *Escherichia coli*, *in vivo*
Instability index	57.22	Stable
GRAVY	−0.720	Hydrophilic
Allergenicity	Probable non-allergen	No
Antigenicity score	1.3413	Antigenic
Solubility	0.989585	Soluble

^*a*^ “–” indicates that no specific remark is applicable for the corresponding parameter.

### Rational structure of SAMEA and effective binding with TLR2

TLR2 plays an indispensable role in the innate immune system, and its activation is instrumental in enhanced antigen presentation and the promotion of adaptive immunity. This crucial interaction initiates a robust immune response, facilitating higher antibody titers and superior protection against infection. The interaction’s strength is mirrored in the binding affinity between the two molecules. Figure 2B presents the results of the secondary structure analysis, showing that the vaccine’s secondary structure consists of 120 helices and 55 strands. To identify the optimal tertiary structure, we utilized pairwise structural similarity to categorize all decoy models. Of these, Model 1 emerged as the premier choice, corresponding to one of the largest structure clusters identified by I-TASSER. Enhancements were applied to the initial vaccine model on the ModRefiner server. The refined structure exhibited a root-mean-square deviation (RMSD) of 1.565 and a TM-score of 0.9660, highlighting notable improvements. A combination of a low RMSD and a high TM-score indicates the refinement process has enhanced the model’s accuracy, which is crucial for better recognition by immune cells. According to the Ramachandran plot post-refinement, 92.4% of the protein residues were in favored regions, while 3.5% and 2.5% were distributed in additionally allowed and generously allowed regions, respectively. Only 1.6% of residues were found in disallowed regions, underscoring the structural integrity of SAMEA. Such integrity is vital for the vaccine’s stability and its efficacious interaction with the immune system, paving the way for a strong immunogenic response. Figure 2C and D illustrate the refined model structure and its corresponding Ramachandran plot. Further investigations revealed the SAMEA model effectively docked with TLR2. It should be noted that TLR2 does not possess a classical enzymatic “active site,” but instead recognizes ligands through its extracellular leucine-rich repeat (LRR) domain. Employing the ClusPro 2.0 server resulted in the formation of 21 clusters, which were then ranked based on their energy level. Among all the docking models produced, the one boasting the lowest energy score of −1,171.6 was selected as the most effective docking complex (Fig. 2E). Interface analysis revealed that SAMEA binds extensively to the extracellular surface of TLR2, and the binding interface is located within a surface region consistent with the known ligand-recognition LRR domain of TLR2. This score is a testament to a strong binding affinity and effective receptor engagement by the vaccine model, emphasizing its potential efficacy.

To accurately assess structural changes during the interaction between SAMEA and TLR2, we employed RMSD. The values initially showed an increase during the first 20 ns of the simulation, reflecting initial structural changes, before gradually stabilizing. This stabilization suggests a stable binding interaction between SAMEA and TLR2, with RMSD values maintaining a narrow range thereafter, indicating minimal impact on the overall protein stability. To further explore protein dynamics, RMSF was calculated to evaluate flexibility across different regions. The results displayed lower RMSF values at the binding interfaces compared to higher values at non-interface regions, highlighting the complex’s stability and flexibility. Additionally, the radius of gyration (Rg) remained stable throughout the simulation, suggesting that the interaction did not significantly alter the protein’s overall compactness and structural integrity.

### Expression and purification of SAMEA

**Fig 3:**
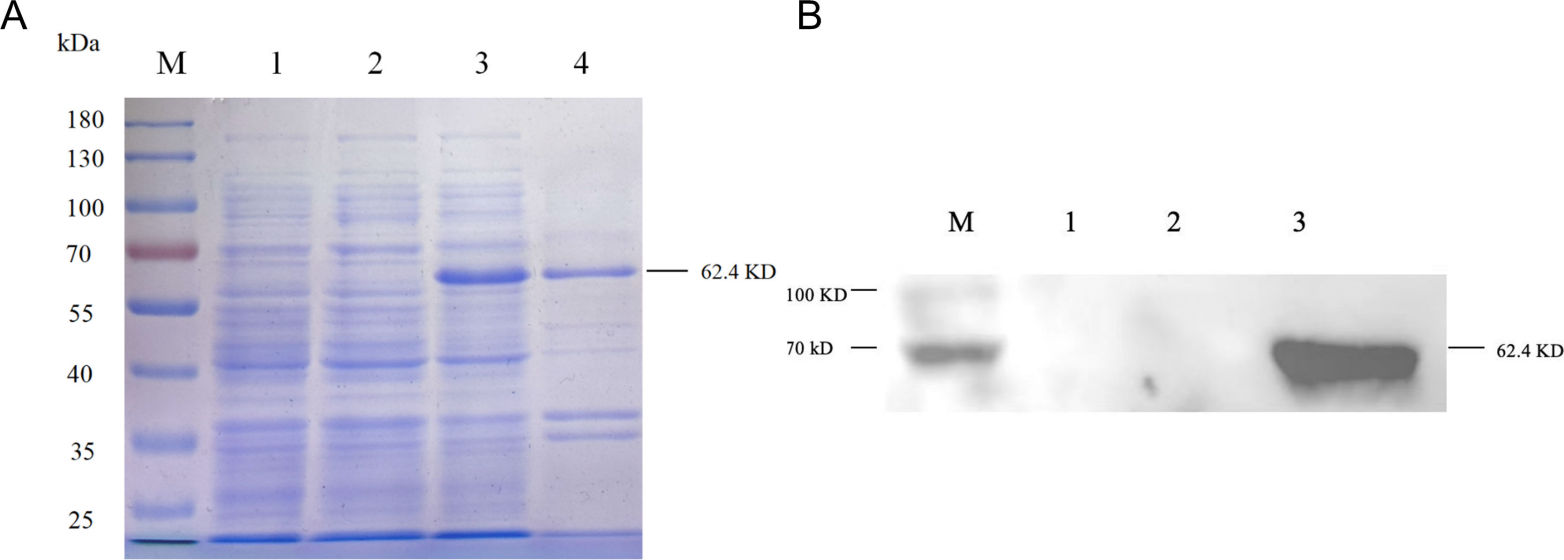
Expression and Western blot analysis of recombinant SAMEA. (**A**) SDS-PAGE analysis of SAMEA expression. Lane M: molecular weight marker; Lane 1: Negative control (nontransformed *E. coli* BL21 cells); Lane 2: Uninduced pET24a-SAMEA/BL21 cell lysate; Lane 3: Soluble supernatant fraction after sonication; and Lane 4: Pellet after sonication (insoluble fraction). (**B**) Western blot identification of SAMEA. Lane M: Protein molecular weight marker; Lane 1: Negative control (*E. coli* BL21 cells); Lane 2: Uninduced pET24a-SAMEA/BL21 cell lysate; and Lane 3: Soluble supernatant fraction containing the induced SAMEA protein.

We successfully expressed and purified the multi-epitope vaccine candidate, SAMEA (Fig. 3). SDS-PAGE analysis revealed a distinct band corresponding to the expected molecular weight of SAMEA, confirming successful expression of the protein. After purification, a single, sharp band was observed on the gel, indicating the effective removal of non-target proteins and the achievement of high-purity protein samples. To further validate the identity of the purified protein, Western blot analysis was performed using a specific antibody (rehabilitation sera) or anti-His antibody against SAMEA. A specific signal band was detected at the position matching the anticipated molecular weight, thereby not only confirming the expression and purification of SAMEA protein but also suggesting its potential applicability in immunogenic studies.

### SAMEA’s initial immunological success: enhanced survival and immune modulation via adjuvant integration

**Fig 4:**
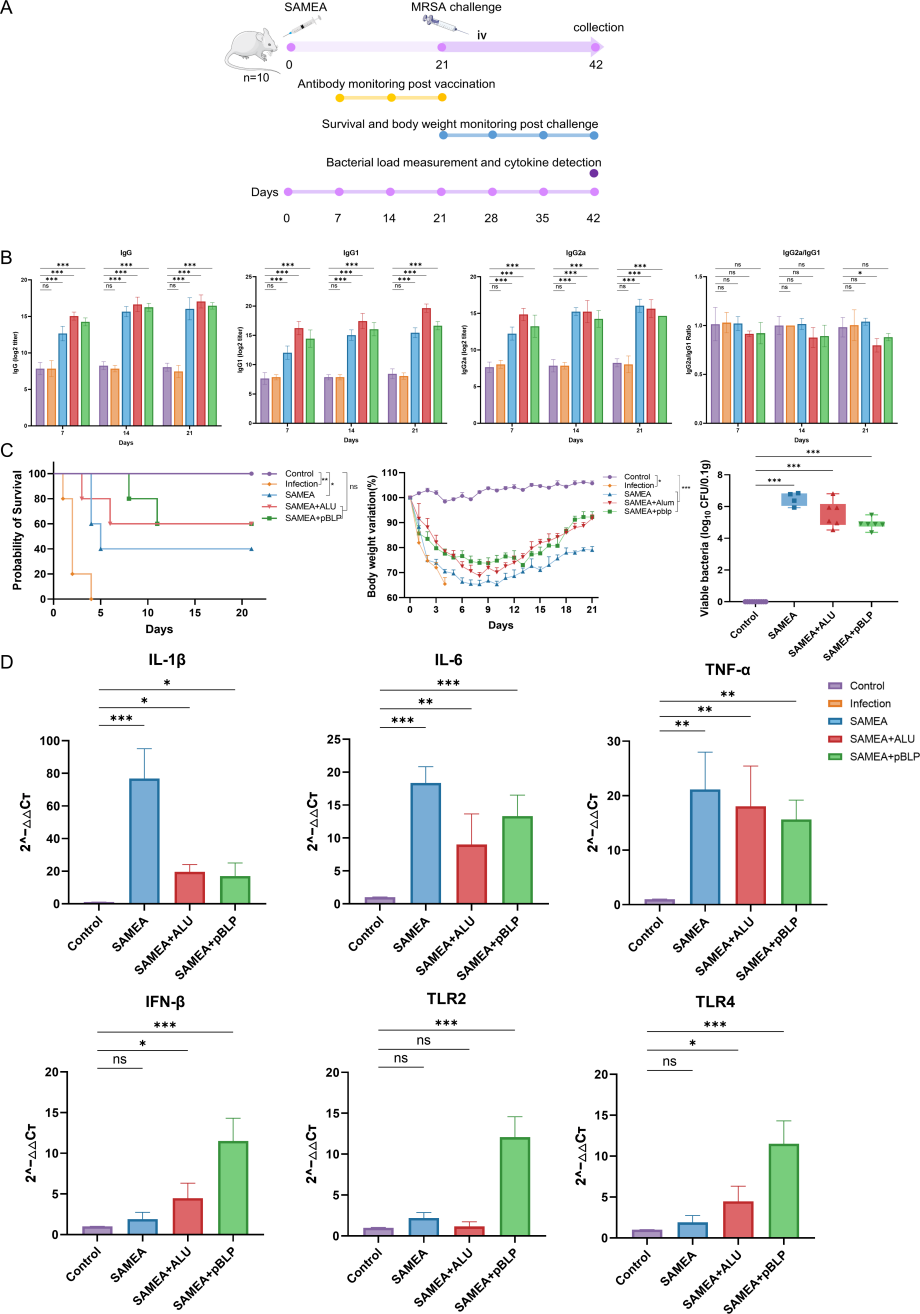
SAMEA with one dose protects mice against challenge with MRSA. (**A**) Schematic diagram of the vaccination and challenge timeline. (**B**) Levels of specific IgG, IgG1, and IgG2a antibodies and the IgG2a/IgG1 ratio (*n* = 10). (**C**) Survival rates, body weight changes, and bacterial loads in kidneys after MRSA challenge (*n* = 10). (**D**) Relative mRNA expression levels of IL-1β, IL-6, IFN-β, TNF-α, TLR2, and TLR4 (*n* = 10). Data are presented as mean ± SD. Significance was determined by one-way ANOVA with Tukey’s post hoc test or Log-rank test (for survival) (*P < 0.05, **P < 0.01, and ***P < 0.001).

To ascertain SAMEA’s immunological efficacy, we developed a mouse model, as depicted in Fig. 4A, incorporating adjuvant alum or pBLP to potentiate SAMEA’s immunological effects. Moreover, all mice were in a good state of clinical health without adverse reactions after immunization. As shown in Fig. 4C, SAMEA significantly bolstered protective efficacy and extended the survival span of mice. Upon *S. aureus* challenge, the survival rate of immunized mice escalated to 60%. Despite an initial decline in immunized mice weight post-challenge, a marked recovery was observed within a week, particularly in the adjuvant-supplemented groups, mirroring enhancements in kidney bacterial clearance. Notably, both alum and pBLP dramatically diminished kidney bacterial burdens, with pBLP demonstrating superior efficacy.

Indirect ELISA assays were utilized to measure SAMEA-specific antibody responses, assessing IgG, IgG1, and IgG2a titers alongside the IgG2a/IgG1 ratio (Fig. 4B). The immunized mice showed significantly higher levels of total IgG, IgG1, and IgG2a at all testing points compared to the control groups. Over a period of 21 days, the levels of all types of antibodies in the immunized group of mice continued to increase, whereas there was no significant change in the levels of antibodies in the control group of mice. The addition of alum and pBLP adjuvants amplified the antibody responses induced by SAMEA. The IgG2a/IgG1 ratios were lower in the SAMEA + alum and SAMEA + pBLP groups. Furthermore, the SAMEA + alum group showed a pronounced decrease, suggesting a potential shift toward a Th2-dominant immune response.

To analyze the efficacy and durability of the immune response, we measured cytokine levels in mice after challenge (Fig. 4D). The results indicated a strong immune response and inflammatory reaction, as evidenced by increases in cytokines IL-1β, IL-6, TNF-α, and TLR4. Although TLR2 expression was increased in the SAMEA group, suggesting an immune response, there was no significant difference compared to the control group. However, the addition of pBLP exhibited a significant increase in TLR2 expression, indicating enhanced immune recognition and activation. This highlights the role of pBLP in modulating the immune response to improve vaccine efficacy. The subtle immunomodulatory effect of alum and pBLP resulted in a balance of inflammatory and protective immune responses, demonstrating the safety and efficacy of SAMEA.

### SAMEA prime-boost strategy: unleashing superior immunoprotection against MRSA

**Fig 5:**
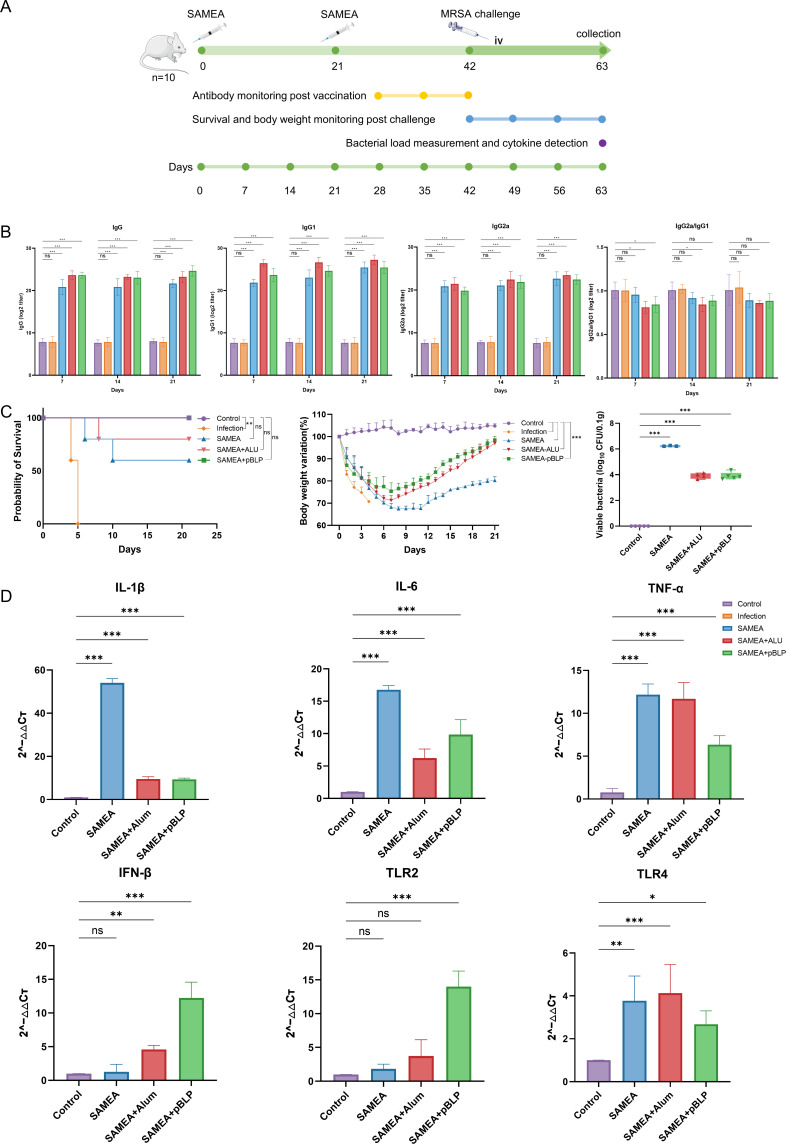
SAMEA in prime-boost strategy protects mice against challenge with MRSA. (**A**) Schematic diagram of the vaccination and challenge timeline. (**B**) Levels of specific IgG, IgG1, and IgG2a antibodies and the IgG2a/IgG1 ratio (*n* = 10). (**C**) Survival rates, body weight changes, and bacterial loads in kidneys after MRSA challenge (*n* = 10). (**D**) Relative mRNA expression levels of IL-1β, IL-6, IFN-β, TNF-α, TLR2, and TLR4 (*n* = 10). Data are presented as mean ± SD. Significance was determined by one-way ANOVA with Tukey’s post hoc test or Log-rank test (for survival) (*P < 0.05, **P < 0.01, and ***P < 0.001).

To investigate the potential of SAMEA to enhance and maintain immunity, a strategic boosting approach was employed after the initial single-dose immunization experiments (Fig. 5A). The survival curves revealed a marked improvement in protective efficacy, showing a 20% increase in survival rates in mice after the second immunization with SAMEA compared to the first. This robust enhancement, particularly when combined with adjuvants, enabled SAMEA to achieve a 100% survival rate in the challenged group, resulting in high protective efficacy. Correlating with these findings, the body weight trends suggested resilience against infection-induced weight loss, highlighting the SAMEA’s efficacy in maintaining normal physiological states post-challenge. Upon further examination of the bacterial counts, a marked reduction was observed, suggesting the booster immunization significantly amplifies the bacterial clearance capacity of the mice, a testament to the SAMEA vaccine’s potential in enhancing the host’s defensive mechanisms.

The impact of the booster dose on antibody levels was significant, with a surge to at least 128 times that of the initial immunization (Fig. 5B). This signifies SAMEA’s ability to heighten the humoral immune response, as illustrated by the significant elevation in IgG, IgG1, and IgG2a levels, further augmented by the strategic use of alum and pBLP adjuvants. SAMEA efficiently promotes a balanced Th1/Th2 response, as demonstrated by the shifting IgG2a/IgG1 ratios. The IgG2a/IgG1 ratio of mice in the SAMEA group decreased after the booster immunization, in contrast to the single immunization. However, the IgG2a/IgG1 ratio of mice immunized with SAMEA and supplemented with alum or pBLP tended to increase. This increase may be due to the nature of the antigens or adjuvants used, or the maturation of the immune response over time. The noticeable increase in antibody profiles highlights the complex regulation of the immune response by SAMEA, demonstrating its flexibility and effectiveness in improving long-term immunity.

Furthermore, cytokine production was evaluated in various groups of mice (Fig. 5D). Results showed that booster immunization led to a decrease in levels of IL-1β and TNF-α, while IL-6, IFN-β, and TLR2 levels remained unchanged. Similar to single immunization, the presence of adjuvant resulted in a reduction of pro-inflammatory cytokine levels. Additionally, a significant decrease in TLR4 expression was observed in the pBLP group after booster immunization, which was approximately one-third of the expression observed after single immunization. However, there was no significant change in TLR4 expression in the SAMEA group or the SAMEA + alum group. In conclusion, booster immunization improved the immunization effect of the subunit vaccine and provided robust protection for the mice.

## DISCUSSION

*S. aureus* is a predominant pathogen causing infections in humans and animals globally. It is associated with a broad spectrum of diseases, ranging from mild to severe, potentially life-threatening conditions (7, 24). The escalating and widespread antibiotic resistance of *S. aureus* highlights the vital importance of developing vaccines as a key strategy to prevent infections caused by this pathogen. In this study, we established a novel multi-epitope vaccine construction strategy, “Just the Best Ones,” and accordingly prepared a novel biomolecule, SAMEA, and showed the construction process and structure. SAMEA has been demonstrated to induce the production of potent antibodies in mice, to modulate the immune response, and has the potential to provide significant protective immunity with a favorable safety profile.

In developing the SAMEA against *S. aureus*, we selected several key antigens based on the pathogenic mechanism of the bacterium in the host, targeting the different phases of infection: adhesion, invasion, and colonization. Critical to establishing an infection, *S. aureus* initially adheres to either biotic or abiotic surfaces (103). Two prominent surface adhesins, ClfA and FnbpA, facilitate this adherence. ClfA, as a cell wall anchoring protein, specifically binds to fibrinogen in the host, promoting *S. aureus* adhesion and aggregation (104). It also acts as a force-sensitive molecular switch that, when subjected to strong mechanical forces, activates adhesion to Fg-conditioned surfaces (57). FnBPA is essential for mediating *S. aureus*’s attachment to fibrinogen and fibronectin (Fn), which is particularly important in wound and medically related infections (59, 105). In addition, IsdB promotes adhesion to and internalization of *S. aureus* by platelets interactionas (62) well as non-phagocytic human cells (106). Plc also contributes by disrupting host cellular membranes, facilitating the spread of infection into and between host cells. Once adhered, *S. aureus* can invade host cells through a variety of mechanisms with the assistance of several factors, resulting in the spread of infection within the host(107). Hla is significant in *S. aureus* invasiveness (108), and antibodies against Hla could neutralize the toxin to reduce the severity of the infection. SpA mediates *S. aureus* invasion across airway epithelial cells by activating TNF receptor 1 and EGF receptor (EGFR) signaling pathways, disrupting the cytoskeleton, and allowing bacterial invasion through paracellular junctions (109). As an essential surface protein involved in the initial interaction with the immune system, SpA is likely to be a key target for the induction of an efficacious immune response. Consequently, it is frequently selected as a crucial antigen for the development of *S. aureus* vaccines. Additionally, MntC plays a critical role in the resistance of *S. aureus* to oxidative stress (110) by competing with host calprotectin for free manganese (111), which is closely related to its immune evasion mechanism. Upon invasion, *S. aureus* proliferate and colonize the biotic or abiotic surface by forming a biofilm. The surface-anchored binding protein sasX, which binds to human matrix molecules known as MSCRAMMs (microbial surface components recognizing adhesive matrix molecules), has been shown to significantly influence *S. aureus* nasal colonization and enhance *in vivo* virulence in various major types of *S. aureus* infections (68). The polysaccharide intercellular adhesion (PIA), also known as poly-N-acetyl glucosamine (PNAG) (112), represents the most well-understood mechanism of biofilm formation and plays a crucial role in the biofilm formation processes of staphylococci, including *S. epidermidis* (113). In addition, FnBPA also promotes biofilm formation in clinically relevant MRSA strains (114). Finally, considering that all superantigenic toxins are strong nonspecific T-cell stimulators (115), we chose SEB as an antigen because SEB contributes to systemic infections associated with cytokine storms (74). In summary, epitopes predicted on the basis of these antigens are expected to elicit protective immune responses against various stages of infection.

In our research, the “Just the Best Ones” strategy is employed for multi-epitope vaccine design, where we focus on selecting the highest-scoring epitopes from each antigen. This method is based on precise bioinformatics tools and algorithms to ensure the selected epitopes exhibit maximal immunogenicity and optimal binding capacity. BepiPred 2.0 employs a combination of sequence analysis and machine learning techniques, utilizing epitope data derived solely from crystal structures for training. It identifies potential B-cell epitopes by analyzing the amino acid composition and surface accessibility of protein sequences. The epitope data predicted by BepiPred 2.0 is considered to be of higher quality, significantly enhancing its predictive capabilities (80, 116). Polyiam et al. (117) employed Bepipred to predict B-cell epitopes in the SARS-CoV-2 ratchet receptor-binding domain (RBD) and a rhesus monkey serum ELISA to validate its efficiency, suggesting that BepiPred is an effective technique. NetCTL 1.2 is used to predict CTL epitopes, analyzing peptide binding to MHC class I, TAP transport efficiency, and proteolytic cleavage sites. It outperforms EpiJen, MAPPP, MHC-pathway, and WAPP across all performance parameters (118). NetMHCIIpan-4.0 beats other cutting-edge algorithms by incorporating NNAlign_MA into the model to predict peptide binding affinity to MHC class II molecules (119). This was also evidenced in Sohail et al.’s study (120), where NetMHCpan-4.0 demonstrated one of the highest hit rates for the SARS-CoV-2 epitope set. Choosing epitopes that score highest on the basis of these predictions, we can efficiently identify candidate epitopes most likely to elicit a strong immune response, as discussed in Ahmad et al. (121), while ensuring that the vaccine design covers a variety of immune response types. The advantage of this method lies in its ability to ensure the immunogenicity and efficacy of the selected epitopes, as each chosen epitope represents the highest score within its respective antigen. In contrast to other multi-epitope vaccine designs (45, 122, 123), which may include a range of generally scoring but potentially immunogenic epitopes, our strategy may be superior in ensuring the efficiency and precision of the selected epitopes. Upon screening, it was discovered that some of the epitopes we predicted and screened overlapped with previously studied ones. Specifically, the B-cell epitopes selected for SpA were found to overlap with the B-cell epitope KKNAFYQVLNMPNLNADQRNGFIQE, identified by Shi et al. (47), that has been shown to stimulate the production of SpA-neutralizing antibodies to protect animals from *S. aureus* colonization and infection. Furthermore, the B-cell epitope TGSNANPTLKETKG and CTL epitope IVLFNRMGGTYIKSG of Plc that we have chosen are identical to those identified by Soltan et al. (124), which has been shown to provide a high level of protection. As a result, our approach to multi-epitope vaccine design provides an efficient way to focus resources and attention on the optimal epitope while maintaining the vaccine design’s comprehensiveness, which is critical for the development of vaccines that can address multiple immune challenges. However, while this strategy is efficient, it may omit epitopes with slightly lower scores but of equivalent importance. Sohail et al. (120) emphasized the importance of comprehensively assessing the diversity of antigenic epitopes to ensure that the vaccine triggers a full immune response. We therefore conducted follow-up animal studies to demonstrate the feasibility of this approach.

Importantly, the construction of SAMEA was not based on a direct concatenation of predicted epitopes, but rather on a structured and hierarchical integration strategy. Candidate epitopes were refined through a consensus-based framework, where predictions from multiple independent tools were cross-validated and filtered using stringent safety criteria. To enhance biological relevance, overlapping or adjacent epitopes derived from the same antigen were merged into continuous peptide regions, reducing redundancy while preserving the native sequence context required for efficient antigen processing and presentation. Building on this curated epitope pool, the “Just the Best Ones” strategy was applied to select only the highest-performing epitopes across different immune categories, ensuring both immunological efficiency and structural compactness of the final construct. These epitopes were then assembled using optimized linkers to maintain structural flexibility and minimize inter-epitope interference. Together, this design framework indicates that SAMEA represents a rationally optimized immunogen rather than a simple aggregation of predicted epitopes, which may underlie its favorable immunogenicity observed *in vivo*.

SAMEA has strong immune-activating properties, particularly in inducing specific immune responses, especially when combined with alum adjuvant or pBLP. Using ELISA, we measured total IgG as well as IgG1 and IgG2a subclasses, and observed significantly increased antibody levels in all SAMEA-immunized groups, consistent with prior reports of antibody responses to *S. aureus* antigens (125–128). Beyond overall IgG induction, IgG subclass profiles provide context for the type of helper milieu engaged. In mice, IgG1 is commonly associated with Th2/IL-4-skewed responses, whereas IgG2a is more often linked to Th1/IFN-γ-associated immunity; thus, the IgG2a/IgG1 ratio can serve as an indirect indicator of Th1/Th2 polarization. Unlike the high IgG2a/IgG1 ratios induced by live-attenuated small-colony variant (*ΔvraGΔhemB*) (129) as well as capsular polysaccharide-protein conjugate (130), the near-balanced IgG2a/IgG1 ratio elicited by SAMEA suggests that the response was not strictly Th2-biased but may involve mixed Th1/Th2 features. Given that effective defense against *S. aureus* often requires coordinated antibody effector functions and cellular immune support, such a response profile could be favorable for protection. Notably, IgG2a may be informative not only as a marker of Th1-associated skewing but also for its potential effector advantages. Previous studies have shown that murine IgG2a interacts more efficiently with Fcγ receptors on macrophages, which can enhance antibody-dependent effector processes, including opsonophagocytosis (123, 131). Because macrophage-mediated clearance is considered an important mechanism in controlling extracellular *S. aureus*, the IgG2a response observed here is consistent with an increased capacity for opsonic clearance. In addition, the IgG2a/IgG1 ratio varied across immunization strategies and time points, consistent with the notion that subclass distributions can be shaped by repeated antigen exposure, adjuvant cues, and dosing schedules. For example, the increase in IgG2a/IgG1 after boosting in the SAMEA + alum group suggests that alum-associated responses may be context-dependent rather than uniformly Th2-skewed, depending on factors such as antigen properties and immunization regimen. Although alum is classically linked to Th2 polarization, Th1-associated readouts have been reported under certain conditions, particularly in combination settings that engage innate pathways. Siram et al. (132) adsorbed novel synthetic TLR4 and TLR7/8 ligands onto aluminum salts and observed enhanced Th1-type immunity, illustrating how formulation context can shift the overall immune profile. Similarly, the distinct trends observed in the SAMEA + pBLP group between single and booster immunizations imply that innate receptor signaling interacts with the immunization protocol to optimize subtype distribution (133). These observations also suggest that epitope composition and antigen presentation may contribute to shaping response quality, aligning with the rationale of our “Just the Best Ones” strategy to prioritize high-performing epitopes. Overall, within the scope of the present data, our findings support the humoral immunogenicity of SAMEA and provide a basis for subsequent cellular-mechanism studies and functional antibody evaluation.

The protective efficacy of SAMEA was evaluated in a *S. aureus* challenge model. Remarkably, while the control mice succumbed to infection, the SAMEA vaccine, particularly when formulated with immune enhancers, conferred robust protection, achieving up to 100% survival in the tested cohort, highlighting the great potential of vaccine formulations to enhance immunogenicity and efficacy. The survival observed here is broadly comparable to protection levels reported for other multi-epitope or subunit candidates. For instance, the vaccine containing B- and T-cell epitopes of Plc studied by Soltan (124) produced 80% protection. Similarly, Als3-Th cell epitopes prepared by Ma et al. (128), when combined with synergistic adjuvants such as CpG, MDP, and FIA, also managed 80% protection against strains such as *S. aureus* Newman and *S. aureus* Wood46. Additionally, the rFSAV vaccine developed by Zeng et al. (35) reached a protection rate of 87%. Together, these comparisons place SAMEA within the range of prior candidates and support further evaluation of our “Just the Best Ones” epitope-selection strategy. Beyond survival rates, clinical indicators further attested to the vaccine’s ability to control infection severity. Although all mice experienced initial weight loss due to acute illness, the immunized groups, especially SAMEA + alum and SAMEA + pBLP, exhibited rapid recovery (Fig. 5C), indicating effective mitigation of systemic toxicity. Crucially, the significant reduction in renal bacterial load in these groups provides direct evidence that the induced immunity effectively restricted bacterial dissemination and colonization in target organs. This clearance capability is consistent with the observed IgG subclass pattern described earlier. The fact that this protection was observed 21 days post-immunization further confirms the durability of the immune memory primed by the vaccine. Collectively, these *in vivo* results establish SAMEA as a highly promising candidate capable of overcoming the limitations of previous formulations.

Following immunization with SAMEA, the host immune system demonstrated an enhanced capacity to combat *S. aureus* infection through the precise regulation of pro-inflammatory cytokines and the upregulation of specific receptors. To further elucidate the protective mechanisms, we monitored the cytokine microenvironment and receptor expression profiles within the kidney. The transcriptional upregulation of IL-1β, IL-6, and TNF-α observed post-challenge indicates the active recruitment and activation of effector immune cells, such as neutrophils and macrophages, to the target organ(134). These pro-inflammatory cytokines serve as key effector molecules of cellular immunity, directly driving the phagocytic activity required for bacterial clearance (68, 111). Notably, we observed significant differences in expression patterns between the SAMEA group and the adjuvant-formulated groups (SAMEA + alum/pBLP). The SAMEA group maintained high levels of inflammatory cytokines, indicating that the antigen itself is capable of triggering and sustaining a robust antimicrobial defense in the infected tissue. However, considering that our sampling time point was day 21 post-challenge, this period reflects the “residual inflammatory burden” following infection control rather than the acute inflammatory peak. Therefore, the lower levels of IL-1β, IL-6, and TNF-α observed in the formulated groups are consistent with a state of “rapid resolution of inflammation.” Combined with the lower renal bacterial load and improved physical recovery in these groups, this pattern suggests that the adjuvant formulations enhanced infection control efficiency. By accelerating pathogen clearance, they reduced persistent PRR stimulation, thereby lowering the subsequent tissue inflammatory burden while ensuring effective antimicrobial defense. This aligns with the immunomodulatory effects of alum observed in other inflammation models (135). Furthermore, the decline in IL-1β and TNF-α levels observed after booster immunization compared to the prime dose reinforces this concept of immune precision. Beyond the classical pro-inflammatory axis, changes in IFN-β provide additional insights into intracellular sensing and immune regulation. Although typically associated with antiviral responses, IFN-β can also be induced by the PRR network during bacterial infections, where it influences the antimicrobial programs and inflammatory balance of myeloid cells. For instance, TLR4-related signaling or intracellular receptors (such as NOD1/NOD2) can participate in IFN-β induction (136), and previous studies indicate that insufficient IFN-β induction during *S. aureus* infection correlates with increased pathogenicity (137). In this study, the elevated IFN-β levels in the formulated groups point to the involvement of PRR-related signaling programs, which likely play a role in balancing antimicrobial defense and inflammation regulation. At the upstream recognition level, the upregulation of TLR2 and TLR4 provides mechanistic support for these effects, with the enhancement of TLR2 being particularly pronounced in the SAMEA + pBLP group. TLR2 is a critical receptor for recognizing gram-positive bacteria; its expression on myeloid cells can drive defense circuits via the MyD88-NF-κB/MAPK axis (138), promoting phagocytic clearance (139). Importantly, the pBLP-induced upregulation of TLR2 may not be limited to myeloid cells. Since B cells can express TLR2 and B-cell-intrinsic MyD88 signaling has been linked to IgG2a/c class switching in T-cell-dependent settings (140), pBLP might contribute to the observed protective phenotype by engaging TLR2-MyD88-associated signaling, potentially influencing both humoral responses and innate antibacterial effector functions. Simultaneously, the upregulation of TLR4 may play a key role in overall immune vigilance. A previous study reported that *S. aureus* is unable to directly activate TLR4 but appears to be regulated by TLR4 and shares commonalities with immune responses induced by gram-negative pathogens (141). As demonstrated in *S. aureus* brain abscess models, deficiencies in either TLR2 or TLR4 are associated with higher mortality and bacterial loads, further supporting the importance of both receptors in infection control (142). In summary, the SAMEA vaccine (especially when combined with adjuvants) establishes a highly efficient and safe immune protective barrier by remodeling receptor recognition thresholds, activating intracellular surveillance pathways, and promoting the rapid resolution of inflammation. Finally, protection was evaluated in relatively small mouse cohorts and against a single challenge strain (MRSA USA300); larger studies and a broader panel of clinical isolates (including additional MRSA and MSSA lineages) are required to establish robustness and cross-protective generalizability. Together, these follow-up studies will refine the vaccination strategy and better define the scope and mechanisms of SAMEA-mediated protection.

In the context of our current research on the SAMEA vaccine, we have established a robust foundation with encouraging *in vivo* results that demonstrate the vaccine’s potential efficacy. However, certain limitations have been identified that require further investigation to fully realize the vaccine’s potential. Currently, our study has not incorporated *in vitro* cell-based assays, which would offer valuable insights into the cellular mechanisms underpinning the vaccine’s action (143). Future work will therefore prioritize cellular confirmation using approaches such as ELISPOT and splenocyte proliferation to strengthen mechanistic claims (144). In particular, assessing Th17-associated responses will be important given their recognized role in host defense against *S. aureus* (145). Moreover, beyond conventional immunogenicity measurements, incorporating functional correlates of protection—such as opsonophagocytic uptake/killing assays (OPA/OPKA)—would enable a more direct assessment of antibody effector function relevant to bacterial clearance and help link immune readouts to protection outcomes. Furthermore, our immunization protocol is currently based on a two-dose regimen. Examining the impact of a three-dose schedule and optimized intervals could clarify the durability and magnitude of immune memory (35, 41, 146). We also acknowledge that protection was assessed using a single challenge strain (MRSA USA300). Although USA300 is a predominant and highly virulent clone, *S. aureus* is genetically diverse. Future studies should evaluate cross-protection against a broader panel of clinical isolates, including additional MRSA and MSSA lineages, to define the generalizability of SAMEA-induced protection. In addition, the protective efficacy of SAMEA was evaluated in mouse models with a relatively limited sample size; larger cohorts will be needed to confirm the robustness and reproducibility of the observed protection. Finally, to address the translational gap from murine models to clinical application, we will extend evaluation to larger animal models to optimize dosing strategies and immune monitoring, and explore adjuvant strategies that are compatible with human use. Collectively, these efforts will clarify mechanisms of protection and more precisely define the scope and durability of SAMEA-mediated efficacy.

Our new multi-epitope vaccine construction strategy, “Just the Best Ones,” which involves selecting multiple antigens and then carefully identifying the best-performing epitope from each antigen, has been validated in mouse models. In response to the lack of an effective vaccine for *S. aureus*, especially MRSA, we used *S. aureus* as a model antigen, developing the multi-epitope vaccine, SAMEA. It was the first attempt at such a vaccine construction strategy, and SAMEA showed outstanding advantages in terms of stability, immunogenicity, and safety, and this approach may achieve better results in clinical trials than past multi-epitope vaccines. These results allow SAMEA to be defined as a promising vaccine candidate to address the growing crisis of *S. aureus* infections, thus demonstrating the great potential of the “best of the best” strategy for multi-epitope vaccine research. As we continue to refine and apply this approach, it will greatly facilitate the development of vaccines against complex pathogens and enable more effective prevention of infectious diseases.

### Conclusion

In this study, we established a new strategy, “Just the Best Ones,” for screening antigenic epitopes for multi-epitope vaccines and demonstrated the effectiveness of this method in a mouse model. On this basis, we developed SAMEA as a highly efficient vaccine candidate against *S. aureus.* SAMEA has excellent immunogenicity and stability, with the potential to improve long-term immunity by increasing antibody levels and modulating the Th1/Th2 immune response, resulting in a strong protective effect.

Our results demonstrate the utility of the “best of the best” strategy and provide a new approach for the development of multi-epitope vaccines. In addition, the SAMEA developed based on this strategy can be used as a potent vaccine candidate for the prevention of *S. aureus* infections, which provides a new idea for the prevention of *S. aureus* infections.

## Data Availability

The data that support the findings of this study are openly available in ScienceDB at https://doi.org/10.57760/sciencedb.14333.

## SUPPLEMENTAL MATERIAL

Data S1 spectrum.03085-25-s0001.docx Includes the final SAMEA sequence, structural validation analyses, and MD-based characterization of SAMEA-TLR2 interactions.

Data S2 spectrum.03085-25-s0002.xlsx ELISA data.

Highlights spectrum.03085-25-s0003.docx Highlights of the manuscript.

Supplemental material spectrum.03085-25-s0004.docx Legends.

ASM does not own the copyrights to Supplemental Material that may be linked to, or accessed through, an article. The authors have granted ASM a non-exclusive, world-wide license to publish the Supplemental Material files. Please contact the corresponding author directly for reuse.
