# Supplementary material for: “Just the Best Ones” combination: a new strategy for multi-epitope vaccine candidate based on immunoinformatics analysis to induce protective immunity against MRSA infection
Source: Microbiol Spectr. 2026 Jul 13;14(8):e03085-25. doi: 10.1128/spectrum.03085-25 (PMC13435805; doi:10.1128/spectrum.03085-25)
Supplement: Supplementary file 1 [file spectrum.03085-25-s0001.docx]

**Supplementary Sequence. The final SAMEA design**. The finalized amino-acid sequence of the SAMEA construct is provided. The sequence represents the final design used for downstream computational analyses and experimental validation.

TGSNANPTLKETKGKKQSSNTNAEELKKNGEETLTSKKKTKGTAKDIIEKKVIFGTPSFIIKKKKNAFYQVLNMPNLNADQRNGFIQEKKHGGGVTDKDNKKYYPRNSIDTKKKKLTDADVILYKKINSHQTDKRKGGGSIVLFNRMGGTYIKSGGGGSSEDEANTSLGGGSFTDYIDYKVGGGSAAHKHVRSKGGGSILGWIFFFFGGGSKADAQQNNFGGGSTTDNNVSTQGGGSLSSGFSPDFGGGSKLVPLLLALGGGSDVFGANYYYGPGPGKSGVRFFDIRGRASAGPGPGSNKDVDSQAVNPSAPGPGPGGGKIRYTFTDYIDYKGPGPGINNKVITYDIGYSYMGPGPGIDDTEINQLKLLYYVGPGPGSGGVTPAANAAQHDEGPGPGDNNVSTQENNTQSTQGPGPGGTTDIGSNTTVKTGDGPGPGDVKPIYLNGEEGNKDGPGPGINVKSIDQFLYFDLI

**Supplementary Figure S1. Chi–Chi2 and Residue property distribution of the predicted SAMEA structure.**

**Supplementary Figure S2. Planarity analysis of the predicted SAMEA structure.**

**Supplementary Figure S3. Structural interface and hydrogen bonding interactions between SAMEA and TLR2 based on the MD-refined complex**
