# Supplementary material for: “Just the Best Ones” combination: a new strategy for multi-epitope vaccine candidate based on immunoinformatics analysis to induce protective immunity against MRSA infection
Source: Microbiol Spectr. 2026 Jul 13;14(8):e03085-25. doi: 10.1128/spectrum.03085-25 (PMC13435805; doi:10.1128/spectrum.03085-25)
Supplement: Supplementary file 2 [file spectrum.03085-25-s0003.docx]

**Highlights**

- Introduced “Just the Best Ones”, a strategy involves the selection of multiple antigens, followed by meticulous identification of the top-performing epitope from each antigen.
- The designed *Staphylococcus aureus* Multi-Epitope Antigen (SAMEA) has optimal structural features, high antigenicity, biocompatibility, and high binding affinity with TLR2.
- SAMEA exhibits safety and immunogenicity in mouse model.
- With the assistance of adjuvants, SAMEA greatly enhanced TLR2 expression and completely protected mice from a lethal dose of MRSA.
