# Supplementary material for: “Just the Best Ones” combination: a new strategy for multi-epitope vaccine candidate based on immunoinformatics analysis to induce protective immunity against MRSA infection
Source: Microbiol Spectr. 2026 Jul 13;14(8):e03085-25. doi: 10.1128/spectrum.03085-25 (PMC13435805; doi:10.1128/spectrum.03085-25)
Supplement: Supplementary file 3 [file spectrum.03085-25-s0004.docx]

**Supplementary Information (SI)**

**"Just the best ones" combination: A new strategy for multi-epitope vaccine candidate based on immunoinformatics analysis to induce protective immunity against MRSA infection**

This material includes:

**Supplementary File 1. The final SAMEA design**. The finalized amino-acid sequence of the SAMEA construct is provided. The sequence represents the final design used for downstream computational analyses and experimental validation.

**Supplementary File 2. ELISA dat**a. Raw ELISA dataset used for analysis, including individual replicate values for each group and time point.

All PDB-format coordinate files associated with this study have been deposited in Science Data Bank (https://doi.org/10.57760/sciencedb.14333). The deposited dataset includes the final SAMEA construct model and the predicted structural models used in downstream analyses, enabling full transparency and reproducibility of the computational structural workflows reported here.
